# Minimally invasive plate osteosynthesis or conventional intramedullary nailing for distal tibial fractures: A cohort study protocol

**DOI:** 10.1097/MD.0000000000021779

**Published:** 2020-08-14

**Authors:** Xin Song, Xun Huang, Maihemuti Yakufu, Bin Yan, Chencheng Feng

**Affiliations:** a Department of Orthopedics, The Second Affiliated Hospital of Army Medical University, Chongqing; b Department of beauty, Guangzhou Deen Medical Plastic and Aesthetic Hospital, Guangdong; c Department of Orthopedics, The Sixth Affiliated Hospital of Xinjiang Medical University; d Department of Orthopedics, The Seventh Affiliated Hospital of Xinjiang Medical University, Xinjiang, China.

**Keywords:** distal tibial fractures, functional score, intramedullary nailing, minimally invasive plate osteosynthesis, protocol

## Abstract

**Background:**

Currently, both minimally invasive plate osteosynthesis (MIPO) and intramedullary nailing are the two most commonly used methods of treatment in distal tibial fractures, but controversy still exists regarding the clinical effects of 2 techniques. Our purposes were to compare MIPO and intramedullary nailing for distal tibia shaft fractures by assessing functional outcomes and complications.

**Methods:**

Data were collected retrospectively from the charts of patients treated for distal tibial extra-articular fractures between May 2012 and July 2018. All cases were performed by a single surgeon. Institutional review board approval in the Second Affiliated Hospital of Army Medical University was obtained prior to conducting chart review and analysis. The criteria for inclusion in the study were being aged at least 18 years at the time of diagnosis and having a closed or type I open fracture of the distal third of the tibial diaphysis. The primary outcome compared between the 2 groups was the American Orthopedic Foot and Ankle surgery score. The secondary outcome measures in this trial included Olerud and Molander Ankle Score, radiographic outcomes, and complications. Statistical analysis was performed using SPSS version. *P* values < .05 were considered statistically significant.

**Results:**

We hypothesized that MIPO would be associated with better functional outcomes and fewer complications.

**Trial registration:**

This study protocol was registered in Research Registry (researchregistry5808).

## 1 Introduction

The distal tibial fractures are the most common bone fractures of the lower extremity, constitute about 10% to 13% of all tibial fractures.^[1,2]^ Distal tibial fractures are often caused by high-energy injury, and the incidence of complications, caused by comminuted fractures and soft tissue damage due to poor blood supply, are higher for distal tibial fractures than for shaft fractures. These fractures can cause substantial disability in patients if no timely and proper treatment is provided.^[3,4]^

The optimal mode of surgical treatment in distal tibial fractures has been an area of debate for decades. The common surgical procedures included open reduction and internal fixation, external fixation, minimally invasive plate osteosynthesis (MIPO), and intramedullary nailing. Distal tibial fractures have historically been treated with open reduction and internal fixation using plates. Although this technique provides predictable reduction quality, it adds the risk of additional soft tissue injury.^[5]^ External fixation can be very useful as a temporary option for skeletal and soft tissue traction, but as a definitive treatment method may result in malunion, non-union, pin tract infection and ankle stiffness.^[6,7]^

Intramedullary nailing is another alternative for the distal tibial fracture. It allows minimally invasive, dynamic fracture fixation and avoids further soft tissue trauma by adhering to the concept of biological osteosynthesis. However, a higher incidence of malunion and anterior knee pain has been common complaints after antegrade tibial nailing in previous studies.^[8–13]^ With the development of minimally invasive technology, MIPO has become an excellent method. It protected the subcutaneous soft tissue of anterior medial tibia and enabled adequate soft tissue coverage overlying the plate with less wound complications.^[14,15]^

Currently, both MIPO and intramedullary nailing are the two most commonly used methods of treatment in distal tibial fractures, but controversy still exists regarding the clinical effects of 2 techniques.^[16–22]^ Some authors argued that intramedullary nailing was superior, while some authors suggested that the MIPO technique provided better functional and clinical results. Therefore, our purposes were to compare MIPO and intramedullary nailing for distal tibia shaft fractures by assessing functional outcomes and complications. We hypothesized that MIPO would be associated with better functional outcomes and fewer complications.

## 2 Materials and methods

### 2.1 Study design and patients

Data were collected retrospectively from the charts of patients treated for distal tibial extra-articular fractures between May 2012 and July 2018. All cases were performed by a single surgeon. Institutional review board approval in the Second Affiliated Hospital of Army Medical University was obtained prior to conducting chart review and analysis (CQ2020093). This study was also registered in the Research Registry (researchregistry5808). The criteria for inclusion in the study were being aged at least 18 years at the time of diagnosis and having a closed or type I open fracture of the distal third of the tibial diaphysis. Exclusion criteria were earlier fractures of the tibial shaft on the same side, proximal intraarticular or distal intra-articular fractures of the tibia, fractures within 6 cm of the ankle joint, and temporary treatment with an external fixator. Trauma radiographs were used to determine the location and AO classification of the fractures in the selected patients (Table 1).

**Table 1:**
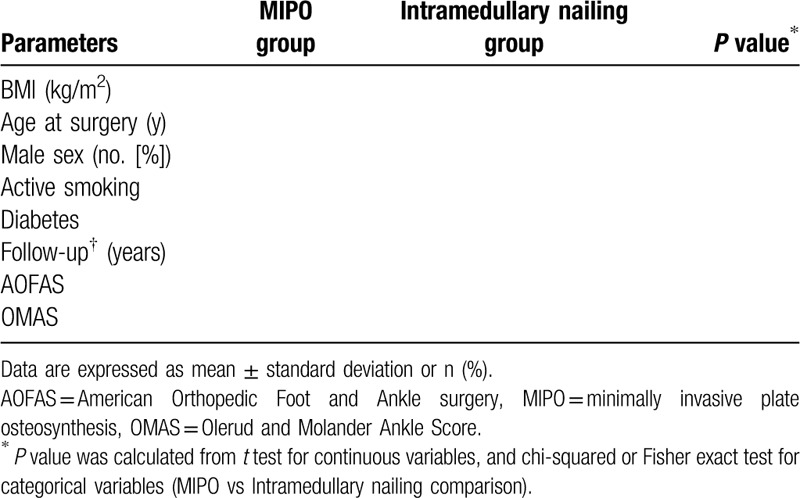
Pre-operative data.

### 2.2 Operative techniques

Patients were operated under spinal anesthesia in supine position on a standard radiolucent table. Prophylactic intravenous antibiotics were administered 15 minutes before skin incision. An image intensifier was used in all the cases to provide fluoroscopic guidance.

#### 2.2.1 MIPO group

In the MIPO group, a distal incision approximately 4 cm in length was made along the anterior border of the medial malleolus. A proximal incision of 3 to 4 cm was then made at the medial surface of the tibial shaft and the level of the most proximal 3 screw holes. An extraperiosteal tunnel was made with a blunt dissection using a periosteal elevator from the distal to the proximal window. Under image intensifier control, reduction was achieved indirectly. The plate position was adjusted when reduction was achieved. No less than 6 cortical layers should be purchased for each side of the fracture.

#### 2.2.2 Intramedullary nailing group

In intramedullary nailing group, an interlocked intramedullary unreamed tibial nail was used in all fractures. Access to the proximal tibia was provided by a transtendinous approach. The starting point was made with an awl, and the nail was inserted in an antegrade manner by hyperflexing the knee. Reduction of the fracture often was achieved with gentle manipulation and traction by an assistant.

### 2.3 Postoperative care

Active range of movements of knee and ankle joint along with quadriceps strengthening exercises were started on the next day of surgery. Weight-bearing was not permitted in either group for 6 to 8 weeks postoperatively. Partial weight-bearing was then permitted. At least 3 bridging cortex calluses on biplanar radiographs and absence of clinical pain with full load-bearing was considered full union. Full load was allowed once radiological union was achieved.

### 2.4 Clinical outcome measures

Clinical assessments were performed at six months, one year, and two years after surgery (Table 2). The primary outcome compared between the 2 groups was the American Orthopedic Foot and Ankle surgery (AOFAS) score. The AOFAS score includes nine items that can be divided into three subscales (pain, function and alignment). Pain consists of one item with a maximal score of 40 points, indicating no pain. Function consists of seven items with a maximal score of 50 points, indicating full function. Alignment consists of one item with a maximal score of 10 points, indicating good alignment. The maximal score is 100 points, indicating no symptoms or impairments.

**Table 2:**
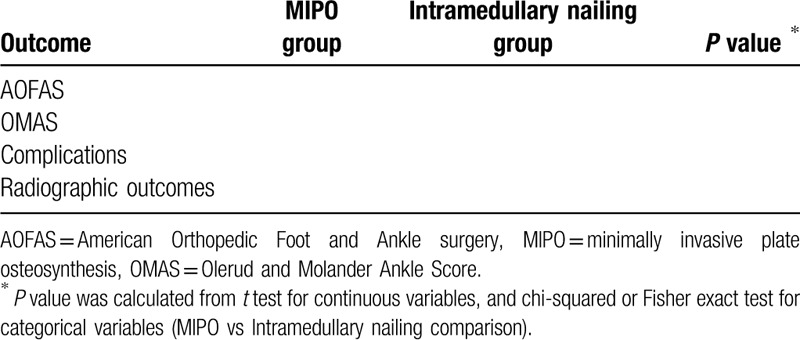
The postoperative outcomes in the 2 groups.

The secondary outcome measures in this trial included Olerud and Molander Ankle Score (OMAS), radiographic outcomes, and complications. The OMAS is a self-administered patient questionnaire. It is a good outcome tool for assessing symptoms after an ankle fracture. The score is based on nine different items: pain, stiffness, swelling, stair climbing, running, jumping, squatting, supports and work/activities of daily living. The scoring system correlates well with parameters considered to summarize the results after this type of injury and is therefore recommended for use in scientific investigations. Radiographs were assessed by a trained reviewer not involved in the patients’ care. Union was defined as healing of at least three of four cortices on biplanar radiographs. Nonunion was defined as lack of any healing within 6 months. Malunion was defined as angular deformity of greater than 4°, translation or shortening greater than 5 mm, or rotational malalignment of greater than or equal to 10°.

### 2.5 Statistical analysis

Statistical analysis was performed using SPSS version. Continuous outcome variables and their difference were tested with parametrical statistical techniques, such as *t* tests, unless the normality test showed a nonparametric distribution of the data, in which case a Mann-Whitney test was used. Survival was analyzed using the Kaplan-Meier survival analysis. Significance for survival was calculated using the generalized Wilcoxon test. Categorical outcome variables were analyzed with the Chi-squared test. *P* values < .05 were considered statistically significant.

## 3 Discussion

Selection of a treatment in cases of unstable distal tibial fractures that do not extend over the joint is still a matter of discussion. Various treatment methods such as open reduction and internal fixation, external fixation, intramedullary nailing and MIPO are described for distal tibial fractures. However, there is still no clear consensus on treatment of distal tibial fractures. Our purposes were to compare MIPO and intramedullary nailing for distal tibia shaft fractures by assessing functional outcomes and complications. We hypothesized that MIPO would be associated with better functional outcomes and fewer complications.

## Author contributions

**Conceptualization:** Maihemuti Yakufu

**Data curation:** Bin Yan

**Formal analysis:** Xin Song, Xun Huang

**Investigation:** Xin Song, Xun Huang

**Methodology:** Xin Song, Xun Huang

**Project administration:** Bin Yan

**Supervision:** Chencheng Feng

**Writing – original draft:** Xin Song, Xun Huang

**Writing – review & editing:** Chencheng Feng
